# Residual Tumour at CT Scan Based on Radiologic Peritoneal Carcinomatosis Index After Optimal Cytoreduction in Advanced Ovarian Cancer: A True Prognostic Factor

**DOI:** 10.3390/cancers17050746

**Published:** 2025-02-22

**Authors:** Alexandra Trelis Blanes, Víctor Lago, Rosario Pérez Martínez, Vicente Belloch Ripollés, Guillermina Montoliu, Pablo Padilla-Iserte, Marta Gurrea, Jose Miguel Cárdenas Rebollo, Santiago Domingo

**Affiliations:** 1 Department of Gynaecologic Oncology, University Hospital La Fe, 46026 Valencia, Spain; victor.lago.leal@hotmail.com (V.L.); pablo_iserte@hotmail.com (P.P.-I.-I.); martagurrea@gmail.com (M.G.); santiago.domingo.delpozo@gmail.com (S.D.); 2 Department of Obstetrics and Gynaecology, Hospital Virgen de los Lirios, 03804 Alcoy, Spain; 3 Medical School, CEU Cardenal Herrera, 46115 Valencia, Spain; 4 Department of Radiology, University Hospital La Fe, 46026 Valencia, Spain; perez_mrosariomar@gva.es (R.P.M.); belloch_vicrip@gva.es (V.B.R.); montoliu_guifor@gva.es (G.M.); 5 Department of Applied Mathematics and Statistics, CEU San Pablo University, 28003 Madrid, Spain; cardenas@ceu.es

**Keywords:** advanced ovarian cancer, optimal cytoreduction, CT scan, residual disease, PCI radiologist, prognosis

## Abstract

Introduction: The maximum residual tumour size after surgery is the most important prognostic factor related to survival in advanced ovarian cancer. This parameter can be subjectively determined by the surgeon at the end of the operation and by a radiologist with a postoperative CT scan. CT scans after optimal cytoreduction can reveal residual/progressive disease in a significant percentage of patients, ranging from 21% to 49%. The aim of this study was to validate the PCI scale for the systematic reading of postoperative CT scans in patients with advanced ovarian cancer and to establish it as a new prognostic marker. Material and Methods: Patients with advanced ovarian cancer (FIGO II-IV), diagnosed between 2007 and 2019 in Hospital La Fe Valencia, in whom cytoreductive surgery was performed (achieving R0 or R1), and in whom a postoperative CT scan was performed between the third and eighth week post-surgery and prior to the start of chemotherapy, were included. Two different radiologists who specialised in gynaecological malignancy performed a blind analysis of the CT scans. They then read the images using the Peritoneal Carcinomatosis Index (PCI) scale, which divides the abdominopelvic cavity into 12 quadrants. Using the Qualitative Assessment (QA) scale, they established the presence or lack of tumour disease in each of these regions, with QA 1–2 being definitely/probably normal, QA 3 indeterminate and QA 4–5 probably/definitely metastatic. Results: This study included a cohort of 117 patients. The radiological study found measurable tumour disease in up to 49% of patients after optimal primary cytoreduction (R0 or R1). There was “substantial agreement” between the results of the two radiologists according to the Kappa analysis (0.624). Both radiologists’ (A and B) findings were related to a significant reduction in both disease-free survival (DFS) and overall survival (OS) in patients with residual disease in the CT scan (QA 4–5) versus those without macroscopic disease (QA 1–3) (*p* < 0.05). Conclusions: The finding of radiological tumour disease on a standardised and systematised postsurgical CT scan prior to the initiation of adjuvant chemotherapy is associated with the prognosis of patients with advanced ovarian cancer.

## Simple Summary

In advanced ovarian cancer, the maximum residual tumour size after surgery has been established as the main prognostic factor related to survival. The oncological outcome after debulking surgery is determined by the surgeon at the end of the procedure according to the residual tumour. Although there is no clear recommendation, a CT scan can be performed prior to the start of adjuvant chemotherapy to determine the presence of residual disease. A few studies have evaluated the outcome of CT as a prognostic marker in patients with advanced ovarian cancer, observing discrepancies between the outcomes according to the surgeon in terms of residual disease. In this paper, we have analysed how performing a systematic reading of postsurgical CT scans in patients with advanced ovarian cancer affects the prognosis of these patients. We studied 117 patients who had a second CT reading performed by two different radiologists using the PCI scale.

## 1. Introduction

Advanced ovarian cancer continues to have the highest mortality rate among gynaecological malignancies. The standard of care is cytoreductive surgery, when complete resection is feasible, and platinum/taxol-based chemotherapy [1]. Primary or interval cytoreduction aims to completely remove the macroscopic disease, making it a complex procedure associated with potential morbidity [2]. Some maintenance therapies are determined by the outcome of surgery [3].

Currently, the recommended method of assessing surgical outcome is based on direct visual evaluation of the abdominal cavity at the end of cytoreductive surgery [4]. However, the maximum residual tumour size after surgery has been established as the most important prognostic factor related to survival in advanced ovarian cancer. The classification distinguishes between R0 (complete resection), R1 (residual tumour < 1 cm) and R2 (residual tumour > 1 cm) [5,6]. Determining the extent of residual disease, particularly in complex anatomical regions such as the retroperitoneum or upper hemiabdomen, might lead to underdiagnosis of the remaining disease at the end of surgery [7].

Although not strongly recommended in clinical practice, computed tomography (CT) scans are often performed after cytoreduction and before the initiation of chemotherapy to assess the presence of residual disease or disease progression. However, few studies have investigated the correlation between residual tumour observed on CT imaging and oncological outcomes following optimal cytoreduction [8,9].

According to a small number of published studies, CT scans after optimal cytoreduction have revealed residual/progressive disease in a significant percentage of patients, ranging from 21% to 49% [10,11,12]. Several theories have been put forward to explain this finding, including rapid tumour growth, postoperative artefacts, surgeon underestimation or radiologist overestimation. In addition, the classification and location of residual disease may have a direct impact on the prognosis of these patients [7].

We previously conducted a preliminary study to investigate the relationship between postoperative CT findings and oncological outcomes in patients with advanced ovarian cancer who underwent optimal cytoreductive surgery [12]. A total of 117 patients were included in the analysis, and CT scans were classified into three categories: no evidence of tumour, suspicious findings or conclusive evidence of residual tumour/progressive disease. The study found that 29.9% of postoperative CT scans showed clear evidence of residual tumour/progressive disease. However, no significant differences in disease-free survival (DFS) or overall survival (OS) were observed between the three groups [12].

In the preliminary study, the CT scans were reviewed by several radiologists without a systematic image reading scale. This might have influenced the absence of differences in DFS and OS. To overcome this obstacle, we designed the current study, in which the images were reviewed by two radiologists who specialised in different gynaecological tumours using a validated scale. The aim of this study was to validate the PCI scale for the systematic reading of postoperative CT scans in patients with advanced ovarian cancer and to establish it as a new prognostic marker.

## 2. Materials and Methods

### 2.1. Eligibility

We performed a retrospective analysis that included all individuals diagnosed with advanced ovarian cancer (stage II–IV according to the International Federation of Gynaecology and Obstetrics classification) or relapsed ovarian cancer at the University and Polytechnic Hospital La Fe in Valencia, Spain, from 2007 to 2019. This analysis specifically focused on patients who underwent cytoreductive surgery (either primary or interval) and achieved complete (R0) or optimal (R1) resection. The exclusion criteria included patients who did not have a computed tomography (CT) scan between the third and eighth week after surgery and before starting chemotherapy in the case of primary surgery or re-stating chemotherapy in the case of interval surgery. The study was approved by the hospital’s bioethics committee under local code SDP-BEV-2018-01.

**Figure 1:**
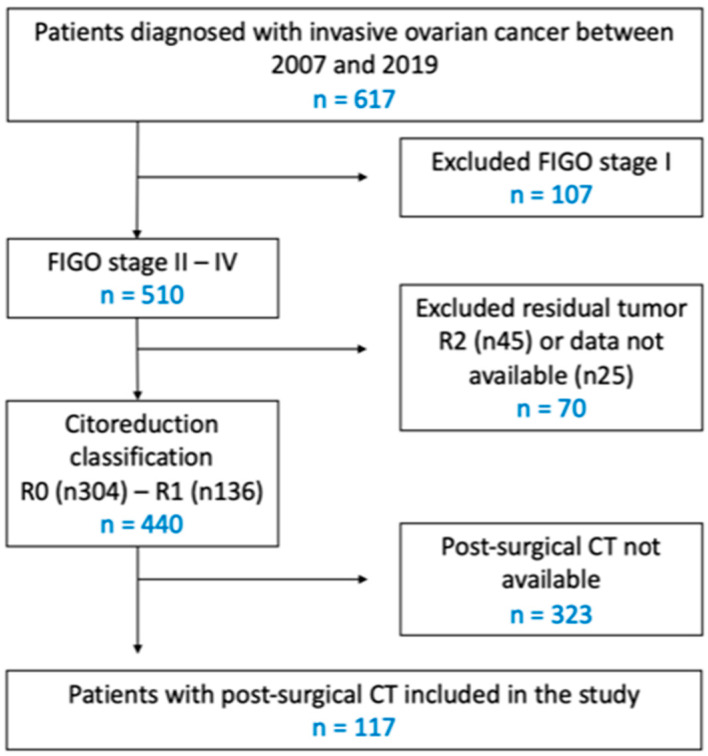
Flow chart.

During this period, surgery was performed on 617 patients with a diagnosis of ovarian cancer. Of these, 510 were classified as having FIGO stage II-IV disease, and of these, 440 patients had a complete/optimal outcome with cytoreductive surgery. A total of 323 patients were excluded from the study because they did not have a CT scan between the third and eighth week after surgery and prior to the start of chemotherapy. Ultimately, the study included a cohort of 117 patients, as shown in Figure 1 (flow chart). The present cohort of patients corresponds to that in our previously published study [12].

### 2.2. Data Collection

The following baseline characteristics were recorded: newly diagnosed or recurrent disease, age at surgery, body mass index (BMI), menopausal status, performance status (ECOG) and neoadjuvant chemotherapy.

The following surgical, postoperative and pathological characteristics were described: presence of ascites, PCI; extended surgical procedures such as bowel resection, lymphadenectomy, omentectomy, diaphragmatic stripping, splenectomy, liver resection and intraoperative transfusion; surgery result (R0 indicating absence of macroscopic disease, and R1 indicating presence of residual disease < 1 cm); days of hospitalisation; complications according to the Clavien–Dindo classification; histotype, binary histology; FIGO stage; BRCA status; total cycles of chemotherapy; days after post-surgery CT and relapse of patients. Relapse confirmed through an imaging test.

### 2.3. Image Analysis and Interpretation

Two different radiologists who specialised in gynaecological disease performed a blind analysis of the CT scans: neither of them had the initial radiological report, only the postsurgical CT images; neither of them were aware of the result reported by the other radiologist. Only the images and the information about the surgical procedure performed were provided. All CT scans selected were optimal for imaging analysis, according to the image contrast, slice thickness, spatial resolution, image noise and absence of artefacts. They then read the images using the Peritoneal Carcinomatosis Index (PCI) scale shown in Appendix A, which divides the abdominopelvic cavity into 12 quadrants. For each of these regions, they used the Qualitative Assessment (QA) scale to establish the presence or lack of tumour disease, with QA 1–2 being definitely/probably normal, QA 3 indeterminate and QA 4–5 probably/definitely metastatic. The CT scan slice thickness used was 5 mm.

### 2.4. Statistical Methods

In the descriptive analysis, quantitative variables are presented as the mean or median ± standard deviation (SD), and qualitative variables are expressed as absolute or relative frequencies. The Kappa index was calculated to determine the concordance between the two radiologists. For the statistical analysis, Kaplan–Meier survival curves were calculated, establishing a 95% CI, and *p* < 0.05 was considered statistically significant. Kaplan–Meier curves were constructed to show the estimated survival prospects. The differences in survival were analysed using the log-rank test. The software used for the statistical analysis was SPSS 20.

## 3. Results

A total of 89.7% of patients (*n* = 105/117) were initially diagnosed with ovarian cancer, while 10.3% (*n* = 12/117) were recurrent cases. The mean age at diagnosis was 55.4 ± 12.3 years and the mean BMI was 25.5 ± 5.2. Regarding performance status (Eastern Cooperative Oncology Group, ECOG), 95.8% (*n* = 112/117) had an ECOG score of 0–1. Cytoreduction prior to chemotherapy was performed in 74.8% (*n* = 88/117) of cases, while interval surgery was performed in 25.2% (*n* = 29/117) of cases.

Appendix A show details of the surgical procedures, postoperative recovery of the patients, and anatomopathological information. Bowel resection, diaphragmatic stripping and splenectomy were performed in 59.5% (*n* = 69/117), 44.4% (*n* = 51/117) and 20.9% (*n* = 24/117) of the patients, respectively, indicating the surgical effort and complexity involved. R0 surgery, defined as the absence of visible tumour remnants at the end of surgery, was achieved in 67.5% (*n* = 79/117) of cases. The remaining 32.5% (*n* = 38/117) of cases resulted in R1 cytoreductive surgery. Serous tumours accounted for 79.3% (*n* = 92/117) of all patients.

An overall Kappa of 0.624 was obtained, which means that the concordance between the two radiologists was substantial. The results of the Kappa analysis to assess the concordance in the results between the two radiologists according to each region of the Peritoneal Carcinomatosis Index (PCI) are shown in Table 1. The Kappa index could not be calculated for regions 11 and 12 due to the low number of events.

**Figure 2:**
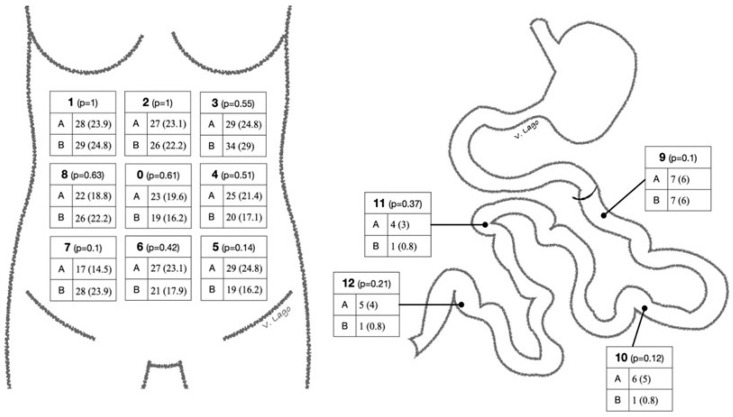
QA 4–5 classification according to PCI for each radiologist (A/B); *n* (%).

The results observed by each radiologist in terms of the number of patients with QA 4–5 (which means probable/definite tumour disease) in each area were analysed. Tumour disease was found in at least one location according to the PCI in 52/117 and 58/117 patients by radiologists A and B, respectively (44 vs. 49.4%; *p* = 0.41). Figure 2 shows the details of QA 4–5 found in each location.

Table 2 shows the prognosis of the patients according to the results of the postsurgical CT report based on the PCI. Based on the results of the scan, the patients were divided into two groups: QA 1–3 (definite/probable/indeterminate absence of tumour) and QA 4–5 (probable/definite presence of tumour).

**Figure 3:**
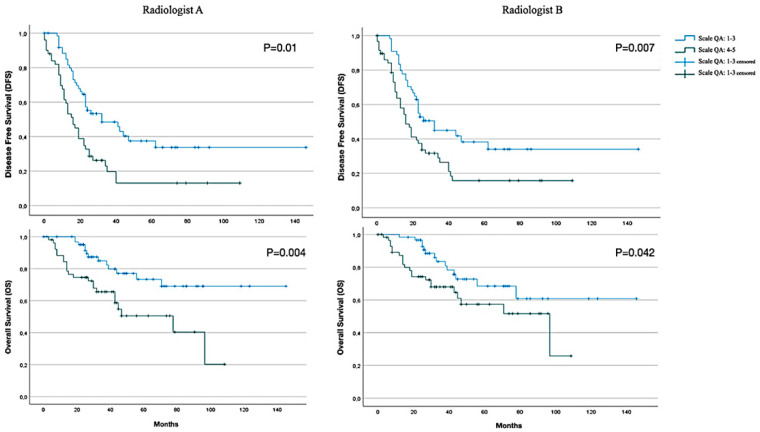
DFS and OS Kaplan–Meier curves.

According to radiologist A’s tumour classification, the mean DFS (disease-free survival, defined as the time that a patient survives without signs of disease after treatment) was 65.3 months (CI 95% 48.4–82.3) for the QA 1–3 group and 28.1 months (CI 95% 17.9–38.2) for the QA 4–5 group. This difference was statistically significant, as shown in the Kaplan–Meier curve in Figure 3 (*p* = 0.01). According to radiologist B, the mean DFS was 65.2 months (CI 95% 47.2–83.2) for the QA 1–3 group and 31.6 months (CI 95% 21.5–41.6) for the QA 4–5 group. This difference was also statistically significant, as shown in the Kaplan–Meier graph (*p* = 0.007).

Regarding radiologist A’s tumour classification, the OS values (overall survival is defined as the length of time from the date of diagnosis to the last follow up or death) were as follows: the mean OS was 112.9 months (CI 95% 97.3–128.5) for the QA 1–3 group and 61.6 months (CI 95% 48.6–74.6) for the QA 4–5 group. As shown in Figure 3, this difference was statistically significant (*p* = 0.004). According to radiologist B’s findings, the mean OS was 105.9 months (CI 95% 87.9–123.8) for the QA 1–3 group and 66.9 months (CI 95% 54.3–79.5) for the QA 4–5 group. As shown in Figure 3, the differences in DFS and OS between the two groups were statistically significant (*p* = 0.042).

## 4. Discussion

### 4.1. Summary of Main Results

In this study, the two radiologists found macroscopic tumour disease in up to 49% of patients after optimal primary cytoreduction, although according to the surgeon, the whole tumour should have been removed (R0) or any remaining tumour should only have measured < 1 cm (R1). Furthermore, there was substantial concordance according to the Kappa analysis between the results of the two radiologists who reported on the CT scans of these patients in a blind fashion.

A statistically significant reduction in disease-free survival (DFS) from 65 months to less than 30 months in patients in whom radiological tumour disease was found (in comparison with those without residual disease), according to the findings of both radiologist A and radiologist B (Figure 3). The same was true in terms of overall survival (OS), with patients without macroscopic disease on postsurgical CT surviving a mean of 112–105 months according to each radiologist’s findings, compared to 61–66 months in patients with persistent disease (Figure 3). Therefore, according to this study, the finding of radiological tumour disease on a standardised and systematised postsurgical CT scan prior to the initiation of adjuvant chemotherapy affects the prognosis of patients with advanced ovarian cancer.

### 4.2. Results in the Context of the Published Literature

The discordance between the surgeon’s assessment of residual disease and that found on radiological imaging may affect the ability to define adjuvant treatment as having had a poor response and, therefore, the patient’s final prognosis.

In our previous study [12], no statistically significant difference was seen in the prognosis of these same patients (Appendix A). This may be due to the fact that in that study, the reading of the postsurgical CT scans was not performed in a standardised manner according to the PCI system, which may have led to subjective impressions on the part of the radiologists.

Some authors [7,8,9,10,11] have concluded that the upper abdomen (corresponding to regions 1, 2 and 3 of the PCI scale) is the most difficult area to explore during surgery, and, coincidentally, in this study, we found that the radiologists classified these areas as Q4–5 in almost 30% of patients.

However, the literature published so far remains contradictory. Chi et al. [10] observed no difference in the DFS or OS of patients with or without residual disease. Eskander et al. [7] concluded that DFS was shorter in patients with residual disease, but this had no impact on OS. Sala et al. [9] and Lorusso et al. [11] found that residual disease on CT scans was associated with a decrease in DFS and OS (Appendix A). Clinical prognostic models integrating postoperative CT scans together with other indicators (preoperative albumin level, age, tumour histology) have also been published, indicating that the radiological presence of residual disease greater than 1 cm in patients with optimal reduction during surgery was associated with an increased risk of disease progression and death [13].

### 4.3. Strengths and Weaknesses

The strength of our study is the systematisation of the postoperative CT analysis with a simple scale that evaluates all areas of the abdomen and allows for less subjectivity on the part of the radiologist. Despite this, our analysis has certain limitations that must be considered. Firstly, the study was retrospective. In addition, there was no histological confirmation of residual disease identified on postoperative CT scan. The size and heterogeneity of the sample must be considered a limitation when interpreting our results. Finally, it is important to note that the performance of each CT scan was at the discretion of the oncologist, as CT scanning prior to the initiation of adjuvant chemotherapy is not yet standard practice in ovarian cancer patients.

### 4.4. Implications for Practice and Future Research

To unify results, it would be necessary to validate a radiological scale such as the one proposed in this study to establish whether it would reduce interobserver variability between radiologists and systematise CT readings.

## 5. Conclusions

The finding on radiological tumour disease on a standardised and systematised postsurgical CT scan prior to the initiation of adjuvant chemotherapy might be associated with a worse prognosis of patients with advanced ovarian cancer.

## Figures and Tables

**Table 1:** Kappa index between radiologist A and B according to Peritoneal Carcinomatosis Index (PCI).

Global 0.624			9 1.00	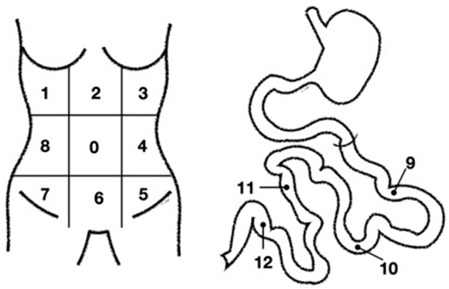
1 0.513	2 0.488	3 0.632	10 0.275	
8 0.634	0 0.421	4 0.534	11 NA	
7 0.485	6 0.530	5 0.533	12 NA	

Strength of concordance: slight (0.01–0.20); fair (0.21–0.40); moderate (0.41–0.60); substantial (0.61–0.80); almost perfect (0.81–1.00); NA: not analysable.

**Table 2:** Oncological outcomes according to postsurgical CT based on PCI findings.

CT report PCI (Radiologist A)	*n* (%)	
Scale QA: 1–3	65 (55.6)	
Scale QA: 4–5	52 (44.4)	
CT report PCI (Radiologist B)	*n* (%)	
Scale QA: 1–3	59 (50.6)	
Scale QA: 4–5	58 (49.4)	
DFS (Radiologist A)	Mean ± DS (95% CI)	Median ± DS (95% CI)
Scale QA: 1–3	65.3 ± 8.6 (48.4–82.3)	32 ± 10.1 (12.2–51.8)
Scale QA: 4–5	28.1 ± 5.2 (17.9–38.2)	16 ± 2.4 (11.2–20.8)
All	50.9 ± 5.9 (39.3–62.6)	26 ± 2.2 (18.7–27.3)
DFS (Radiologist B)		
Scale QA: 1–3	65.2 ± 9.2 (47.2–83.2)	32 ± 4 (24.1–39.8)
Scale QA: 4–5	31.6 ± 5.1 (21.5–41.6)	16 ± 2.4 (11.2–20.8)
All	50.9 ± 5.9 (39.3–62.6)	23 ± 2.2 (18.7–27.3)
OS (Radiologist A)		
Scale QA: 1–3	112.9 ± 7.9 (97.3–128.5)	NA
Scale QA: 4–5	61.6 ± 6.6 (48.6–74.6)	78 ± 21.7 (35.5–120.5)
All	92.6 ± 7.8 (77.4–108)	97 ± NA (NA–NA)
OS (Radiologist B)		
Scale QA: 1–3	105.9 ± 9.1 (87.9–123.8)	NA
Scale QA: 4–5	66.9 ± 6.4 (54.3–79.5)	97 ± 29.7 (38.6–155)
All	92.6 ± 7.8 (77.4–108)	97 ± NA (NA–NA)

DFS: disease-free survival; OS: overall survival; NA: not analysable.

**Figure A1:**
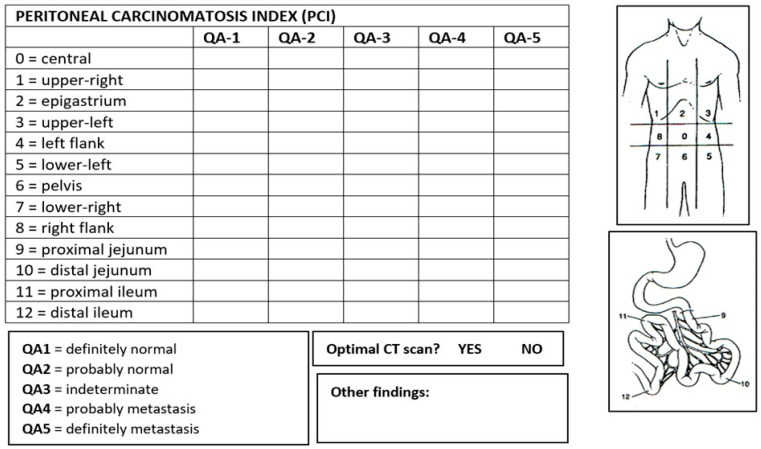
Peritoneal Carcinomatosis Index (PCI) and Qualitative Assessment (QA).

## Data Availability

The data are unavailable due to privacy or ethical restrictions. All results are available in the manuscript and the tables and images provided.

## Appendix A

**Table A1:** The basal characteristics of the patients.

	*n* (%)/Median (DS)
Disease status	
Initial diagnosis	105 (89.7)
Relapse	12 (10.3)
Age	55.4 (12.3)
BMI *	25.5 (5.2)
Menopause	
Pre	37 (31.6)
Post	80 (68.4)
ECOG	
0	89 (76.1)
1	23 (19.7)
2	5 (4.3)
CA 125	895.4 (1710.1)
Neoadjuvant chemotherapy	29 (25.2)

* BMI: body mass index.

**Table A2:** Surgical, postoperative and pathological characteristics.

	*n* (%)/Median (DS)
Ascites (mL)	972.2 (2016.3)
Sugarbaker	12.7 (7.9)
Bowel resection	69 (59.5)
Lymphadenectomy	65 (56.5)
Omentectomy	106 (92.2)
Diaphragmatic stripping	51 (44.4)
Splenectomy	24 (20.9)
Liver resection	5 (4.4)
Intraoperative transfusion	67 (58.3)
Surgery result	
Complete (R0)	79 (67.5)
Optimal (R1)	38 (32.5)
Days of hospitalisation	10.1 (8.5)
Clavien–Dindo complications	
I	18 (15.5)
II	51 (44)
III	16 (13.8)
IV	2 (1.7)
V	1 (0.9)
Histotype	
Serous	92 (79.3)
Endometrioid	6 (5.2)
Mucinous	2 (1.7)
Clear cell	4 (3.5)
Other ^a^	12 (10.3)
Binary histology	
Not Available	5 (4.3)
Low Grade	9 (7.7)
High Grade	103 (88)
FIGO stage	
II	7 (6)
III	87 (75)
IV	22 (19)
BRCA status	
Negative	51 (43.6)
BRCA 1/2	14 (12)
Not available	52 (44.4)
Total number of chemotherapy cycles	
<6	6 (5.3)
≥6	108 (94.7)
Postoperative CT: days after surgery	40 (15)
Relapse	81 (69.2)

FIGO: International Federation of Gynaecology and Obstetrics; ECOG: Eastern Cooperative Oncology Group. ^a^ Including mixed tumours, undifferentiated tumours, germinal tumours, granulosa, carcinosarcoma and germinal tumours.

**Table A3:** Summary of published evidence.

Author (Year)	*n*	Discordance Between Surgeons—CT Scan	DFS Concordant vs. Discordant	OS Concordant vs. Discordant
Chi et al. (2007) [8]	78	39 %	Not analysed	Not analysed
Chi et al. (2010) [10]	67	43 %	21 vs. 17 (*p* = 0.356) ^	60 vs. 43 (*p* = 0.146) ^
Sala et al. (2010) [9]	51	31 %	Not analysed	HR 3.40 (95% CI, 1.4–8.2) (*p* = 0.006) *
Lorusso et al. (2014) [11]	64	20.3%	28 vs. 5 (*p* = 0.001) ^ HR 8.87 (95% CI 3.2–24.3) (*p* < 0.0001) *	Not analysed
Eskander et al. (2018) [7]	627	40 %	18.3 vs. 12.8 (*p* = 0.0059) ^	HR 0.99 (95% CI 0.8–1.2) *
Trelis et al. (2022) [12]	117	29.9%	39.8 vs. 32.8 (*p* = 0.158) ^	67.4 vs. 68.6 (*p* = 0.215) ^
Present study PCI (2023)	117	RxA: 44.4%	65.3 vs. 28.1 (*p* = 0.01) ^	112.9 vs. 61.6 (*p* = 0.004) ^
		RxB: 49.4%	65.2 vs. 31.6 (*p* = 0.007) ^	105.9 vs. 66.9 (*p* = 0.042) ^

CT: computed tomography; DSF: disease-free survival; OS: overall survival; ^ Mantel–Cox (log-rank test). * Multivariate analysis.
